# A Randomized Pilot Trial Assessing the Role of Human Fibrinogen Concentrate in Decreasing Cryoprecipitate Use and Blood Loss in Infants Undergoing Cardiopulmonary Bypass

**DOI:** 10.1007/s00246-022-02866-4

**Published:** 2022-03-19

**Authors:** Christopher F. Tirotta, Richard G. Lagueruela, Apeksha Gupta, Daria Salyakina, David Aguero, Jorge Ojito, Kathleen Kubes, Robert Hannan, Redmond P. Burke

**Affiliations:** 1 The Heart Program, Nicklaus Children’s Hospital, Miami, FL USA; 2 Nicklaus Children’s Health System Research Institute, Miami, FL USA; 3 Dept. Anesthesiology, The Heart Program, Nicklaus Children’s Hospital, 3100 SW 62nd Avenue, Miami, FL 33155 USA

**Keywords:** Cardiopulmonary bypass, Cardiac surgical procedures, Child, Fibrinogen, Hemostasis, Infant

## Abstract

The objective of this study was to determine whether treatment with human fibrinogen concentrate decreases the need for component blood therapy and blood loss in neonate and infant patients undergoing cardiopulmonary bypass. Pediatric patients (*N* = 30) undergoing elective cardiac surgery were randomized to receive human fibrinogen concentrate or placebo following cardiopulmonary bypass termination. The primary endpoint was the amount of cryoprecipitate administered. Secondary endpoints included estimated blood loss during the 24 h post-surgery; perioperative blood product transfusion; effects of fibrinogen infusion on global hemostasis, measured by laboratory testing and rotational thromboelastometry; and adverse events. No clinically significant differences were identified in baseline characteristics between groups. A significantly lower volume of cryoprecipitate was administered to the treatment group during the perioperative period [median (interquartile range) 0.0 (0.0–0.0) cc/kg vs 12.0 (8.2–14.3) cc/kg; *P* < 0.0001] versus placebo. No difference was observed between treatment groups in blood loss, laboratory coagulation tests, use of other blood components, or incidence of adverse events. FIBTEM amplitude of maximum clot firmness values was significantly higher among patients treated with human fibrinogen concentrate versus placebo (*P* ≤ 0.0001). No significant differences were observed in post-drug HEPTEM, INTEM, and EXTEM results. Human fibrinogen concentrate (70 mg/kg) administered after the termination of cardiopulmonary bypass reduced the need for transfusion with cryoprecipitate in a neonate and infant patient population.

*ClinicalTrials.gov identifier*: NCT02822599.

**Supplementary Information:**

The online version contains supplementary material available at 10.1007/s00246-022-02866-4.

## Introduction

Severe postoperative bleeding is a serious complication after cardiac surgery and is associated with increased morbidity and mortality [1]. The bleeding is influenced by both surgical factors and impaired hemostasis. Impaired hemostasis after cardiac surgery may be caused by enhanced fibrinolysis, platelet dysfunction, and coagulopathy, secondary to exposure of blood to artificial surfaces and surgical trauma [2].

Bleeding after cardiac surgery is further complicated in children ≤ 12 months of age with congenital heart disease, as their fibrinogen is qualitatively dysfunctional [3]. Furthermore, the coagulation systems of neonates and children experiencing cardiopulmonary bypass (CPB) are greatly affected by both hemodilution [4]and consumption of blood coagulation factors [5]. For example, CPB has a significant negative impact on rotational thromboelastometry (ROTEM) values in pediatric patients, including those associated with fibrinogen levels [6]. As a result, neonates and young infants undergoing complex cardiac repairs often receive large volumes of blood products, including in the intensive care unit (ICU).

Fibrinogen replacement therapy is currently recommended for both congenital [7] and acquired fibrinogen deficiency caused by various conditions, including major bleeding and cardiac surgery [8–10]. Fibrinogen supplementation can be provided by transfusion of fresh frozen plasma (FFP), cryoprecipitate, human fibrinogen concentrate (HFC), or a combination of the three. HFC has some distinct advantages including viral inactivation, standardized fibrinogen concentration, low infusion volume, no cross-matching required, and ease of reconstitution [11]. Although adverse events (AEs) including thrombosis have been reported in patients treated with HFC, the risk appears to be low [12, 13]. Similarly, thromboembolic events were equally reported in adult patients receiving fibrinogen concentrate or cryoprecipitate after cardiac surgery [14], as well as in pediatric patients, although these were not considered related to fibrinogen concentrate [15]. Indeed, a recent international consensus statement reported that HFC does not appear to increase adverse outcomes, including thromboembolic events, although conclusive evidence is lacking [16].

HFC has been used in the high-risk pediatric patient population at our institution since April 2013. The aim of this study was to determine whether the use of HFC can decrease cryoprecipitate transfusion, blood loss, and the need for component blood therapy in neonates and infants undergoing CPB; safety of HFC was also examined.

## Materials and Methods

This prospective, randomized, placebo-controlled pilot study was approved by the Research Institute of Nicklaus Children’s Hospital and by the Western Institutional Review Board (August 22, 2016; 20140614). Written informed consent was obtained from the parents/legal guardians of participants prior to study entry.

The study was conducted between June 1, 2017 and October 3, 2018 (NCT02822599) at Nicklaus Children’s Hospital, Miami, FL, USA. Neonates and infants (≥ 37 weeks gestational age to 12 months) scheduled for elective cardiac surgery were eligible for inclusion. Exclusion criteria were known prior anaphylactic or severe reaction to the study drug or its components, and FIBTEM maximum clot firmness (MCF) > 15 mm.

Patients (*N* = 30) were randomized (1:1) to either treatment or placebo. The pharmacy performed randomization using Research Randomizer, with patient assignment based on a randomly generated even (treatment) or odd (placebo) number.

The treatment group received a prophylactic infusion of 70 mg/kg HFC (RiaSTAP®, CSL Behring, Marburg, Germany), immediately after CPB termination; the placebo group received normal saline 0.9% (NS) during the same period (NS volume calculated to correspond to that used if the patient was receiving HFC). Both groups received normal standard of care before and after surgery. All operating room (OR) and ICU personnel were blinded to the patient’s group assignment. Only pharmacy personnel not involved in patient care had access to group assignment identity from time of randomization until 24 h post-surgery.

### Endpoints

The primary efficacy endpoint was the amount of cryoprecipitate administered. Secondary endpoints included were as follows: estimated blood loss during 24 h post-surgery; type and total volume of perioperatively transfused blood products; hemoglobin concentration at 2 and 24 h post-surgery; and effects of fibrinogen infusion on global hemostasis at 2 and 24 h post-surgery (prothrombin time [PT], partial thromboplastin time [PTT], international normalized ratio [INR], plasma fibrinogen concentration, platelet count, and ROTEM analysis).

Other secondary endpoints included were number of postoperative hours requiring ventilator support; length of stay in the hospital; length of stay in the cardiac ICU; and re-exploration for bleeding within the first 12 h.

Clinical AEs were defined as any clinical signs of central or peripheral thromboembolism, respiratory or circulatory failure, or allergic reactions during hospital stay. Patients were assessed for signs of thromboembolism every 8 h, per routine care, with each event evaluated by the safety team. Stopping criterion was a statistically significant increase in thromboembolism between the two study groups. All patients were monitored for AEs until discharge.

### Anesthetic Management

Anesthesia was induced with 2 mg/kg propofol (Fresenius Kabi, Lake Zurich, IL, USA), or 5 mcg/kg fentanyl (Hospira, Lake Forest, IL, USA) if the patient had an indwelling intravenous catheter. Otherwise, inhalational induction with sevoflurane 5% (Abbott Laboratories, Abbott Park, IL, USA) was performed. Anesthesia was maintained with sevoflurane and dexmedetomidine (Pfizer, New York, NY, USA; 1 mcg/kg/h). Following anesthetization and intubation, a Foley catheter was placed along with an arterial line and a double lumen central venous line.

### CPB Management

After aortic and atrial purse string sutures were placed, 300 IU/kg heparin (Pfizer, New York, NY, USA) was administered directly into the right atrium to maintain an activated clotting time (ACT) > 400 s. After CPB, 1 mg protamine (Fresenius Kabi, Lake Zurich, IL, USA) for every 100 IU of residual heparin activity was used for heparin reversal to an ACT of < 130 s. A standard non-pulsatile CPB technique with moderate hypothermia (bladder temperature 28–30 °C) or deep hypothermia (bladder temperature 18–20 °C) and hemodilution was used depending on the surgical repair. Cardioprotection was achieved with antegrade cold blood cardioplegia. Weaning-off CPB was performed after rewarming to a bladder temperature of at least 36 °C. The Hepcon® Hemostasis Management System Plus (Medtronic, Inc., Minneapolis, MN, USA) was used to optimize and monitor heparin and protamine dosing.

All patients were < 10 kg in body weight and had the CPB pump primed with one unit of packed red blood cells (PRBC). Target hemoglobin while on CPB was 9 g/dL; the transfusion trigger for additional units of PRBC was 7 g/dL. Patients < 3.5 kg or with an antithrombin III deficiency preoperatively also had one unit of FFP added to the prime. One partial unit of plateletpheresis (200 mL) was administered while rewarming prior to separation from CPB in all patients.

### Drug Protocol and Transfusion Algorithm

Baseline laboratory measurements and an electrolyte profile were obtained pre-surgery. An ACT and thromboelastometry analysis (ROTEM®; Pentapharm GmbH, Munich, Germany) were performed after arterial line placement.

**Fig. 1:**
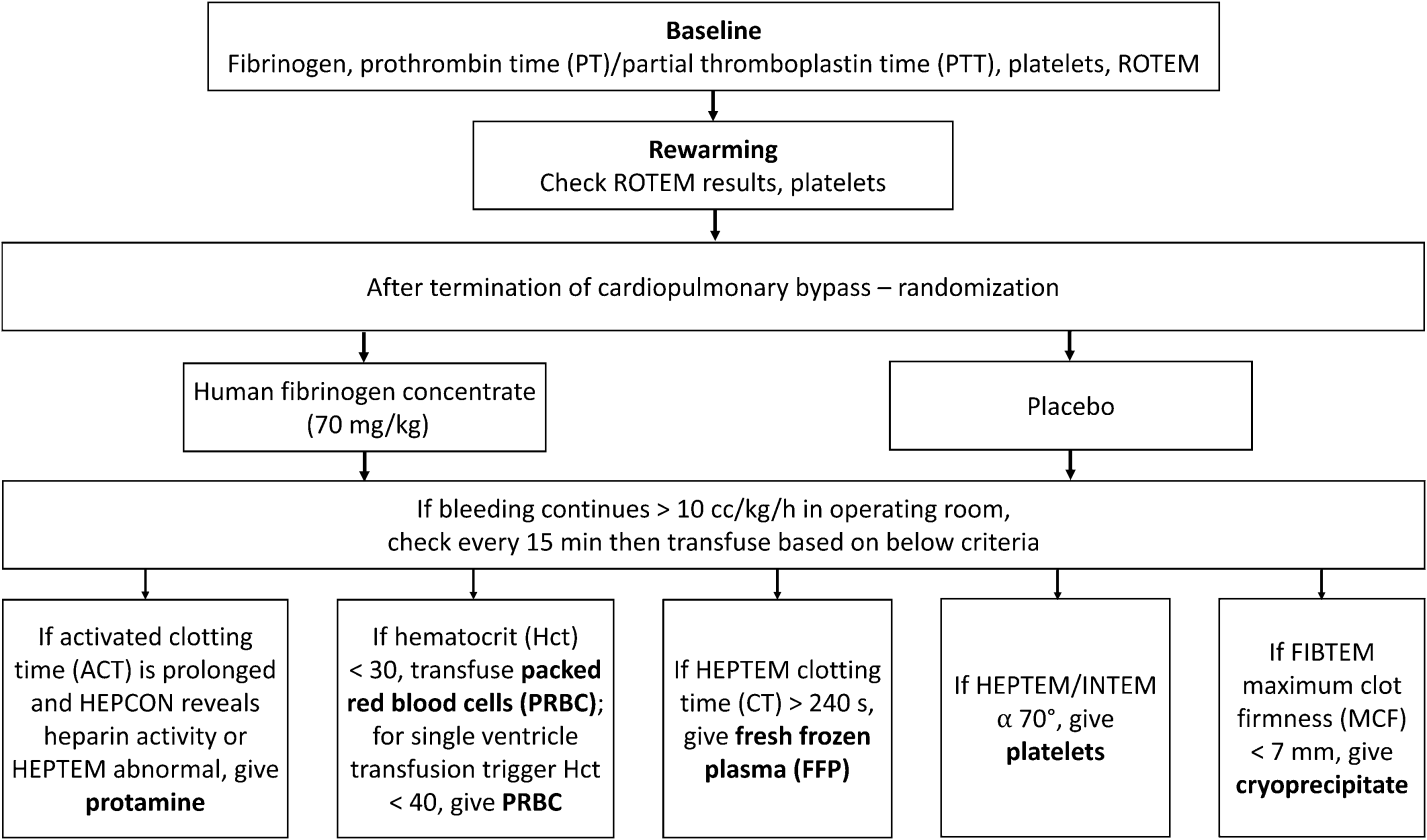
Transfusion algorithm. *ROTEM* rotational thromboelastometry

A transfusion algorithm was developed based on three previous studies, modified for pediatrics [17–19]. Clinically significant bleeding requiring treatment was defined as a rate > 10 cc/kg/h calculated every 15 min while in the OR, measured by blood in the suction canister. In cases of continued bleeding following randomization to HFC or placebo, cryoprecipitate was considered for fibrinogen < 200 mg/dL or FIBTEM MCF < 7 mm (Fig. 1).

Thereafter, as per routine care, ROTEM analysis was performed at least every 30 min while in the OR, or sooner, at the discretion of the OR team. Once the patient left the OR, ROTEM was performed at least every 60 min for the first 6 h or until clinically significant bleeding had stopped.

Blood products transfused after CPB separation were based on a pre-defined protocol. Recommended volumes of each therapy were based on pre-defined formulae and a ROTEM analysis algorithm (Supplementary Table 1 and 2).

If clinically indicated, or to verify treatment effect and control heparin reversal, ROTEM analysis was performed during wound closure. ACT was tested in all patients to control for heparin reversal. If patients had both a pathological ACT and ROTEM, additional protamine was administered. Postoperative bleeding in the ICU was defined as the total amount of chest tube drainage after closure of the sternum and during the first 24 postoperative hours. If the sternum was not closed, it was defined as the total chest tube drainage during the first 24 postoperative hours.

### Statistical Analysis

Descriptive statistics were used to summarize baseline, clinical, and post-drug clinical characteristics. Due to non-normality of the data, *P* values were reported using Exact Wilcoxon 2-sample test to compare ROTEM clinical characteristics between groups for continuous outcomes. For categorical outcomes, Chi-square or Fisher exact test was used to report *P* values. Fisher exact test was used when cell counts were less than five.

To determine if there was a significant difference in FIBTEM between groups with time, repeated measures using mixed model were conducted adjusting for Society of Thoracic Surgeons-European Association for Cardio-Thoracic Surgery (STAT) score and weight. Differences in LS mean effect with 95% confidence intervals (CIs) and Bonferonni-adjusted *P* values were reported for each group, each timepoint, and interaction of group and timepoints to observe any significant differences between groups.

Statistical analyses were performed using SAS Enterprise Guide 7.1 (SAS Institute Inc, Cary, NC, USA) at 0.05 level of significance. There was an insignificant amount of missing data points which had no material consequence on the results.

## Results

### Patient Characteristics

**Fig. 2:**
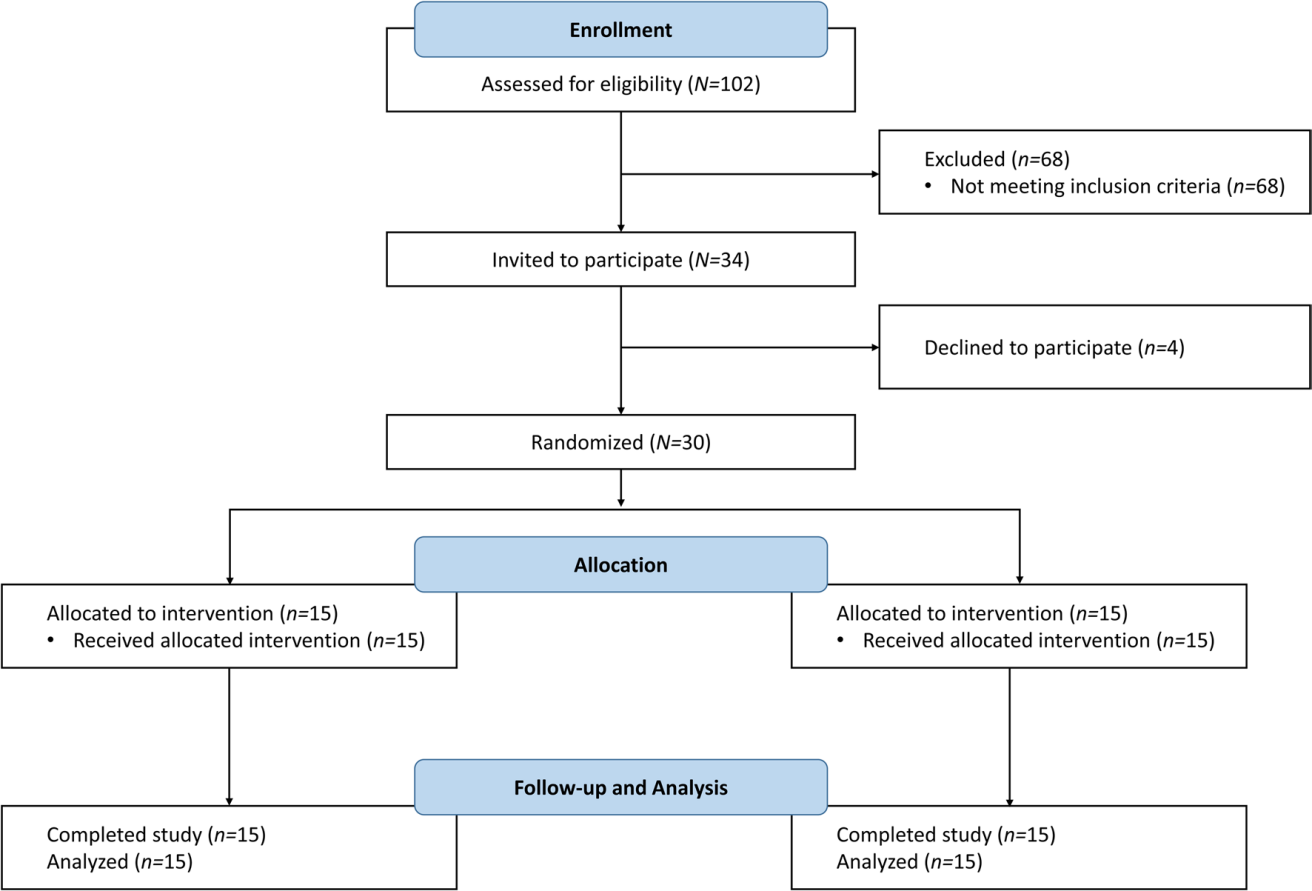
CONSORT diagram

Overall, 30 neonate and infant patients were included (Fig. 2), the majority of whom were white (76.7%) and male (56.7%). Baseline characteristics were well balanced, with no clinically significant differences observed between groups, including both laboratory measurements and ROTEM results (Table 1; Supplementary Table 3). The mean (range) age was 148 (5–286) days and 146 (4–353) days for the treatment and placebo groups, respectively (Table 1).

**Table 1:** Baseline demographic and clinical characteristics

	Treatment		Placebo		*P* value*
	*N*	Mean (Range) or %	*N*	Mean (Range) or %	
Age (days)	15	148 (5–286)	15	146 (4–343)	1.00
Weight (kg)	15	5.3 (3.2–7.3)	15	5.4 (2.8–8.2)	0.86
Height (cm)	15	60 (51–67)	15	59 (47–74)	0.39
Stat Score	15	2.3 (1–5)	15	2.2 (1–4)	0.34
Gender					
Male	9	60.0%	8	53.3%	0.71
Female	6	40.0%	7	46.7%	
Race					
Mixed Race	2	13.3%	0	0.0%	0.4770
Black/African American	3	20.0%	2	13.3%	
White	10	66.7%	13	86.7%	
Ethnicity					
Hispanic Latino	6	40.0%	10	66.7%	0.14
Non-Hispanic Latino	9	60.0%	5	33.3%	
Surgical type					
Ventricular/atrial septal defect	5	33.3%	4	26.7%	0.4846
Arterial switch operation	0	0.0%	3	20.0%	
Norwood stage 1	2	13.3%	0	0.0%	
Bidirectional cavopulmonary anastomosis	3	20.0%	4	26.7%	
Tetralogy of Fallot repair	2	13.3%	3	20.0%	
Central shunt	1	6.7%	0	0.0%	
Aortic arch reconstruction	1	6.7%	1	6.7%	
Repair anomalous right pulmonary artery	1	6.7%	0	0.0%	
Clinical data					
Bypass time (min)	15	128 (52–286)	15	148 (54–414)	0.51
Cross clamp time (min)	15	73 (0–176)	15	89 (0–291)	0.41
Regional low flow perfusion (min)	15	29 (0–194)	15	26 (0–181)	1.00
Deep hypothermic circulatory arrest (min)	15	1 (0–15)	15	0 (0–0)	1.00
Low temperature (°C)	15	27.3 (16.0–34.3)	15	26.1 (15.1–33.4)	0.40
Preoperative lab measures					
Hemoglobin (g/dL)	15	13.6 (11.2–18.0)	15	13.3 (9.7–18.9)	0.71
Hematocrit	15	40.9 (34.9–54.0)	15	39.5 (28.7–53.6)	0.60
Platelets (10 k/uL)	15	341 (188–495)	15	322 (142–515)	0.59
Prothrombin time (s)	15	15.0 (13.9–16.0)	15	15.2 (13.2–16.5)	0.43
International normalized ratio	15	1.2 (1.1–1.3)	15	1.2 (1.0–1.4)	0.26
Partial thromboplastin time (s)	15	38 (29–50)	15	39 (31–49)	0.61
Fibrinogen (mg/dL)	15	227 (164–322)	15	199 (150–285)	0.10
Antithrombin III (%)	15	93 (79–120)	15	89 (58–120)	0.88

*P < 0.05 for continuous outcomes are based on Exact Wilcoxon 2-sample test and for categorical outcomes are based on Chi-square or Fisher exact test

### Efficacy

Significantly less cryoprecipitate (*P* < 0.0001) was given in the treatment group compared to placebo (Table 2; Supplementary Table 4). Moreover, after CPB termination, univariate analysis suggested that FIBTEM MCF values post-study drug administration were significantly different between the treatment and placebo groups (*P* < 0.0001) (Table 3). No significant differences were observed in other ROTEM assays including INTEM, HEPTEM, and EXTEM between the two groups (Supplementary Table 5).

**Table 2:** Post-drug clinical characteristics

	Treatment		Placebo		*P* value*
	*N*	Median (IQR)	*N*	Median (IQR)	
ICU estimated blood loss (cc/kg)	15	35.2 (20.6–61.1)	15	34.4 (27.8–54.3)	0.62
Total Factor VII (mcg/kg)	14	0 (0–0.0)	15	0.0 (0.0–0.0)	N/A
Total packed red blood cells (cc/kg)	15	55.3 (44.7–77.2)	15	57.9 (44.8–89.2)	1
Total fresh frozen plasma (cc/kg)	15	0.0 (0.0–23.2)	15	13.6 (0.0–45.1))	0.27
Total platelets (cc/kg)	15	61.3 (50.1–69.3)	15	59.9 (50.9–73.0)	0.98
Total cryoprecipitate (cc/kg)	15	0.0 (0.0–0.0)	15	12.0 (8.2–14.3)	** < 0.0001**
Total cell saver (cc/kg)	15	27.3 (20.3–34.8)	15	23.7 (13.5–47.5)	0.51
Total blood (cc/kg)	15	142.1 (137.6–174.8)	15	140.9 (136.2–219.3)	0.87
Intubation time (h)	15	0.0 (0.0–28.5)	15	24.0 (0.0–70.0)	0.40
LOS ICU (days)**	14	8.0 (4.0–23.0)	15	8.0 (5.0–12.0)	0.89
LOS hospital (days)**	14	9.0 (6.0–23.0)	15	8.0 (6.0–12.0)	0.75

Bold value denote statistical significance at the *P* < 0.05 level

*ICU* intensive care unit, *IQR* interquartile range, *LOS* length of stay

*P < 0.05 based on Exact Wilcoxon 2-sample test; **excluded a patient with LOS 99 days

**Table 3:** Post-drug FIBTEM results after termination of cardiopulmonary bypass

	Treatment		Placebo		*P* value*
	*N*	Median (IQR)	*N*	Median (IQR)	
Post-drug					
FIBTEM clotting time (s)	15	78.0 (75.0–85.0)	15	94.0 (79.0–129.0)	0.0537
FIBTEM α (°)	11	61.0 (59.0–63.0)	1	66.0 (79.0–66.0)	0.4167
FIBTEM MCF (mm)	15	11.0 (10.0–12.0)	14	6.5 (4.0–9.0)	** < 0.0001**
24 h postoperative					
FIBTEM ICU clotting time (s)	15	60.0 (58.0–67.0)	14	60.5 (58.0–67.0)	0.6099
FIBTEM ICU α (°)	15	72.0 (69.0–77.0)	13	75.0 (72.0–78.0)	0.1643
FIBTEM ICU maximum clot firmness (mm)	15	20.0 (15.0–21.0)	14	22.5 (18.0–24.0)	0.2638

Bold value denote statistical significance at the *P* < 0.05 level

*ICU* intensive care unit, *IQR* interquartile range

*P < 0.05 based on Exact Wilcoxon 2-sample test

**Fig. 3:**
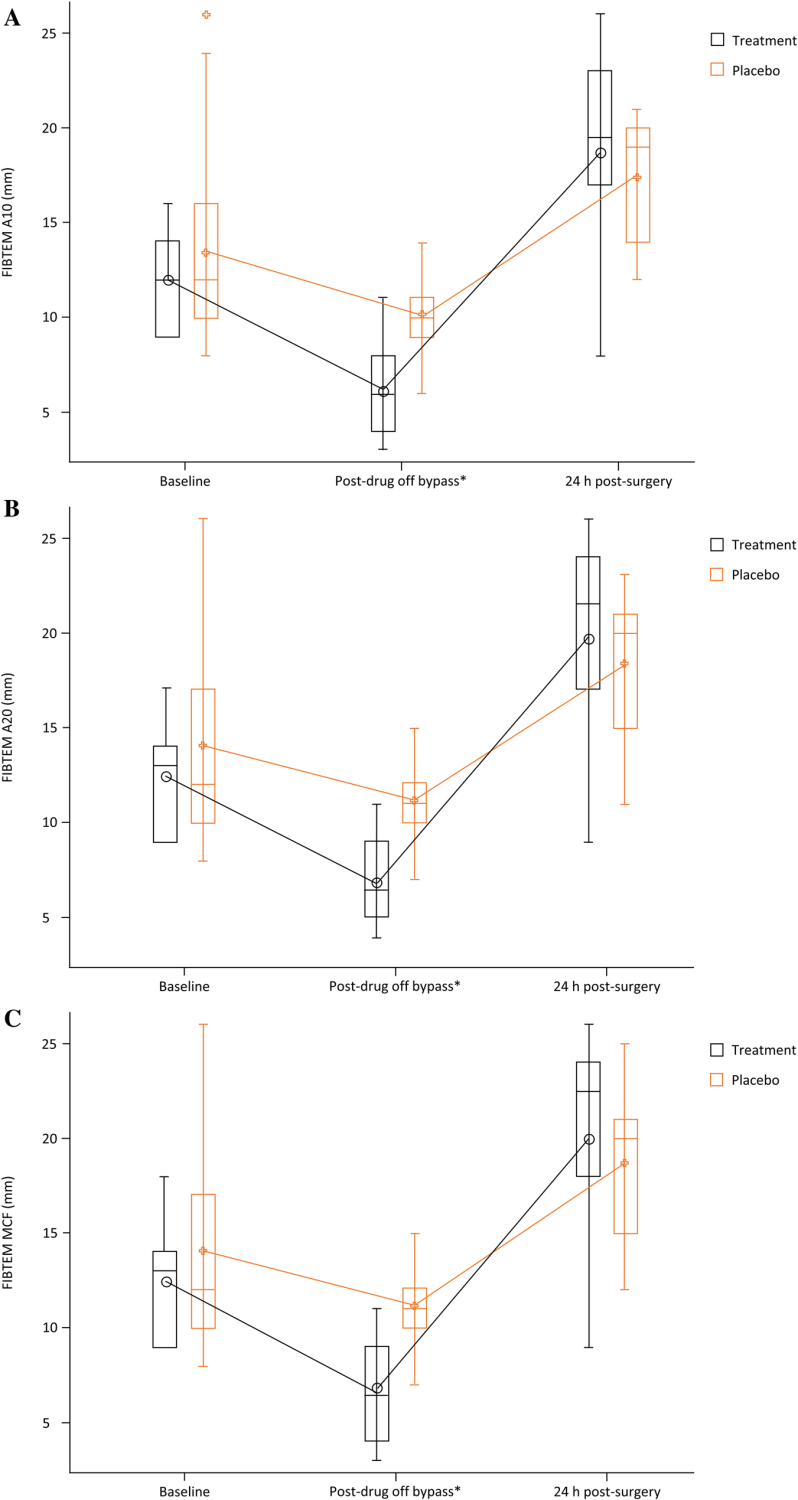
Adjusted FIBTEM graphs for FIBTEM A10 (**A**), A20 (**B**), and MCF (**C**). *Denotes a statistically significant difference between placebo and the treatment group. Whiskers indicate the maximum and minimum range, boundaries of the box indicate 25th–75th percentiles, border within the box indicates the median, ○ or + symbols linked by lines indicate the mean, ○ or + symbols outside the range indicate outliers or single data points. *A10/A20* amplitude at 10/20 min, *MCF* maximum clot firmness

Adjusting for patient’s STAT score and weight suggested that there was a significant difference observed between timepoints after study drug administration in FIBTEM values, including clotting time (CT), amplitude at 10 and 20 min (A10 and A20), and MCF (*P* < 0.05). Similarly, adjusting for STAT score and weight, there were significant changes observed in FIBTEM, including A10, A20, and MCF between groups with time (Table 4, *P* < 0.05; Fig. 3). However, no differences were observed 24 h postoperatively between the two groups.

**Table 4:** Mixed model adjusted for STAT score and weight

ROTEM			
FIBTEM	LS mean effect estimates (95% CI)	Bonferroni-adjusted *P* value	Overall *P* value*
Clotting time (CT)			
Group (Treatment vs Placebo)	− 10.97 (− 22.24, 0.29)	0.0558	0.0558
Changes from time 1 vs time 2	− 43.93 (− 65.58, − 22.29)	** < 0.0001**	** < 0.001**
Changes from time 1 vs time 3	− 6.16 (− 9.58, − 2.75)	**0.0003**	
Changes from time 2 vs time 3	37.77 (16.45, 59.09)	**0.0003**	
Group × time 1	1.67 (− 5.95, 9.29)	1.0000	0.1043
Group × time 2	− 35.80 (− 88.47, 16.88)	0.5650	
Group × time 3	1.21 (− 7.79, 10.21)	1.6000	
α			
Group (Treatment vs Placebo)	− 0.26 (− 4.48, 3.95)	0.8954	0.895
Changes from time 1 vs time 2	6.65 (− 0.61, 13.91)	0.0786	**0.0304**
Changes from time 1 vs time 3	− 2.29 (− 7.27, 2.69)	0.7137	
Changes from time 2 vs time 3	− 8.94 (− 17.03, − 0.85)	**0.0279**	
Group × time 1	− 6.06 (− 2.66, 14.78)	0.4460	0.0454
Group × time 2	− 5.10 (− 23.02, 12.82)	1.0000	
Group × time 3	− 1.75 (− 8.70, 5.21)	1.0000	
Clot formation time (CFT) (s)	NA		NA
A10 (mm)			
Group (Treatment vs Placebo)	1.05 (− 0.90, 3.00)	0.2780	0.278
Changes from time 1 vs time 2	4.57 (2.46, 6.68)	** < 0.0001**	** < 0.0001**
Changes from time 1 vs time 3	− 5.40 (− 8.02, − 2.78)	** < 0.0001**	
Changes from time 2 vs time 3	– 9.96 (– 12.52, – 7.41)	** < 0.0001**	
Group × time 1	1.16 (− 4.29, 6.61)	1.0000	**0.0414**
Group × time 2	3.63 (0.75, 6.51)	**0.0057**	
Group × time 3	− 1.63 (− 6.89, 3.63)	1.0000	
A20 (mm)			
Group (Treatment vs Placebo)	0.71 (− 1.29, − 2.72)	0.4727	0.4727
Changes from time 1 vs time 2	4.02 (2.25, 5.78)	** < 0.0001**	** < 0.0001**
Changes from time 1 vs time 3	− 5.76 (− 8.49, − 3.10)	** < 0.0001**	
Changes from time 2 vs time 3	− 9.81 (− 12.39, − 7.24)	** < 0.0001**	
Group × time 1	0.73 (− 4.74, 6.21)	1.0000	**0.0101**
Group × time 2	3.72 (0.70, 6.74)	**0.0070**	
Group × time 3	− 2.32 (− 7.48, 2.84)	1.0000	
Maximum clot firmness (MCF) (mm)			
Group (Treatment vs Placebo)	1.12 (− 0.80, 3.05)	0.2421	0.2421
Changes from time 1 vs time 2	4.33 (2.04, 6.03)	**0.0001**	** < 0.0001**
Changes from time 1 vs time 3	− 6.17 (− 8.99, − 3.35)	** < 0.0001**	
Changes from time 2 vs time 3	− 10.51 (− 13.28, − 7.74)	** < 0.0001**	
Group × time 1	1.24 (− 4.37, 6.84)	1.0000	**0.0428**
Group × time 2	3.91 (0.71, 7.09)	**0.0077**	
Group × time 3	− 1.78 (− 7.24, 3.67)	1.0000	
Maximum lysis (ML) (%)			
Group (Treatment vs Placebo)	− 0.55 (− 2.60, 1.49)	0.5871	0.587
Changes from time 1 vs time 2	− 2.57 (− 5.34, 1.20)	0.0770	**0.0132**
Changes from time 1 vs time 3	0.72 (− 2.03, 3.48)	1.0000	
Changes from time 2 vs time 3	3.30 (0.50, 6.09)	**0.0155**	
Group × time 1	− 1.19 (− 6.14, 3.77)	1.0000	0.8568
Group × time 2	0.07 (− 4.98, 5.11)	1.0000	
Group × time 3	− 0.54 (− 5.63, 4.54)	1.0000	

Bold values denote statistical significance at the *P* < 0.05 level

Mixed model was used, adjusting for weight and STAT score; time 1 is Baseline, time 2 is post-drug off-bypass, time 3 is 24 h

*A10* amplitude at 10 min, *A20* amplitude at 20 min, *CI* confidence interval, *NA* not available

*Significance *P* < 0.05; significant *P* values in bold. CFT was not collected at second timepoint

### Safety

Overall, 7 (46.6%) and 5 (33.4%) patients from treatment and placebo groups, respectively, had at least one AE. There was no difference in the incidence of clinical AEs between groups, including thromboembolic phenomena (Table 5).

**Table 5:** Adverse events

Adverse events	Treatment *N* (%)	Placebo *N* (%)
Thrombus		
No	11 (73.3)	13 (86.7)
Yes	4 (26.7)	2 (13.3)
Respiratory failure		
No	12 (80.0)	12 (80.0)
Yes	3 (20.0)	3 (20.0)

No significant differences were observed between groups

## Discussion

In this study, prophylactic use of 70 mg/kg HFC significantly improved FIBTEM values and reduced the need for transfusion with cryoprecipitate in neonate and infant patients undergoing CPB. However, there was no change in transfusion requirements with other blood products, including FFP. This is contrary to our previously published retrospective analysis, in which we compared 50 patients who received HFC (70 mg/kg) with 50 age-, diagnosis-, and procedure-matched patients who were treated prior to the introduction of HFC at our institution. Our previous study demonstrated a statistically significant reduction in the need for FFP as well as cryoprecipitate with HFC, without an increase in thromboembolic events [20]. However, the present study had a much smaller sample size (30 patients), compared with 100 patients in the retrospective study, which could explain this result.

Fibrinogen concentration is an independent predictor of postoperative bleeding volume, and patient fibrinogen levels are increasingly monitored using ROTEM, to assess fibrinogen deficiency during surgery. A meta-analysis of 20 publications assessing 5972 patients found an association between lower preoperative and postoperative fibrinogen levels and increased blood loss in cardiac surgery in adults [21]. These results suggest fibrinogen supplementation may be beneficial in patients with a low preoperative fibrinogen level. Similarly, a retrospective analysis of 156 children undergoing cardiac surgery with CPB reported that low plasma fibrinogen levels post-CPB were associated with significant postoperative blood loss [22].

A strong correlation between fibrinogen level and ROTEM MCF has previously been reported [23]. In a study of pediatric patients, CPB induced profound perturbations in ROTEM values, with the most severe changes in neonates and young infants. For every 70 mg/kg of HFC administered to these patients, ROTEM MCF increased by 2.7 mm and fibrinogen concentration increased by 73 mg/dL [6]. Furthermore, for every 38 mL/kg of platelet transfusion, FIBTEM MCF improved by 2.9 mm and HEPTEM α improved by 22.1°. Indeed, our study found FIBTEM MCF significantly improved by ~ 4 mm following HFC administration compared with placebo at the post-drug off-bypass timepoint. Interestingly, no significant difference was observed between treatment and placebo groups in FIBTEM MCF at other timepoints. A likely reason for these results is that the greater use of cryoprecipitate in the placebo group compared with the treatment group led to an improvement in the FIBTEM assay at the later timepoints. Despite the improvement in FIBTEM MCF with HFC, in view of our finding that there was no significant reduction in blood loss with administration of HFC 70 mg/kg versus placebo, it remains unclear what the optimal target fibrinogen level should be in pediatric patients post-CPB.

Use of ROTEM FIBTEM measurements to guide HFC administration to target a high-normal fibrinogen concentration has been associated with a reduction in transfusion requirements, as well as decreased postoperative bleeding, in patients undergoing complex aortic valve operations and reconstruction of the ascending aorta [17]. However, HFC efficacy in cardiac surgery studies varies. A meta-analysis of eight randomized controlled trials in cardiac surgery found that while HFC was associated with a reduction in the incidence of PRBC transfusions, no difference was observed in mortality or other clinical outcomes compared with control [24]. In our study, we found no change in blood loss or PRBC transfusion with HFC administration.

In the large, prospective, multicenter REPLACE study in adults undergoing elective aortic surgery, the use of HFC was associated with an increase in transfusion requirements compared with placebo, even though the FIBTEM target of 22 mm was achieved [25]. The authors attributed these results to factors including low bleeding rates, low plasma fibrinogen concentrations that would not trigger therapy in clinical practice, and variability in adherence to the transfusion algorithm [25]. In the REPLACE study, transfusions occurred at a rate that was three times higher in patients where the algorithm was not followed compared with when it was. As mentioned, the coagulation system of neonates and infants is known to be immature and is profoundly affected by CPB. Critical illnesses are known to disrupt the hemostatic system of neonates and infants, altering the balance of procoagulant and anticoagulant factors, thereby predisposing them to subsequent hemorrhagic or thrombotic complications [26]. The dissimilar patient populations of the REPLACE study and ours could explain the differing results between studies. However, the present study is consistent with results from the FIBRES trial, which demonstrated that HFC was as effective as cryoprecipitate in treating hypofibrinogenemia during surgery [14].

Few studies have evaluated HFC efficacy during cardiac surgery in pediatric patients. In one study, infants undergoing cardiac surgery were randomized to HFC or cryoprecipitate; patients received significantly fewer blood transfusions in the HFC group compared to the cryoprecipitate group [27]. In another study, children (< 7 years) undergoing cardiac surgery were randomized to HFC or cryoprecipitate. There were no differences in blood transfusion or 48 h blood loss between groups [28]. Findings from these studies suggest that HFC is a feasible treatment option in pediatric cardiac surgery and may be a good alternative to cryoprecipitate; however, further studies are required to confirm these findings.

Increases in thromboembolic phenomena were not noted in this trial, nor were they reported in our retrospective analysis [20]. These results are consistent with other studies that reported on the safety of HFC administration. For example, in a pharmacovigilance study, only 28 possible thromboembolic events were reported in > 27 years of surveillance [13]. In addition, no difference in thromboembolic events was observed between HFC and comparator in the meta-analysis described above, with the caveat that studies with adequate power are required to measure this parameter. It should be noted that the small sample size in our current study does not allow for definitive conclusions with respect to AEs. HFC does possess some advantages over cryoprecipitate. These include smaller volumes, negligible chance of a blood-borne infection, and a known and specific amount of HFC in mg. Also, HFC never contains citrate, so there is no risk of acute hypocalcemia and the resultant hypotension.

Several steps were included in the study protocol to minimize the potential for bias. For example, the identity of the study drug may have been discernible after a review of ROTEM values. Therefore, a biostatistician blinded to treatment assignment analyzed the study data independently of the investigator. Based on the post hoc power analysis, the effect of total cryoprecipitate was 0.7, and the power of the study was low (1-β = 50%). Therefore, the main limitation of this study was the low patient numbers and lack of HFC dose ranges tested. As this was a single-center study, the number of available patients for participation in this trial was limited. However, these results demonstrate the potential for treatment with HFC in this patient population. Further studies, including a prospective trial with greater patient numbers, would be warranted and would provide an avenue for future research in pediatric cardiac surgery patients to confirm the results of this study. Additionally, a dose response study to test higher doses in this patient population would provide valuable guidance for clinical use of this therapy. Moreover, due to the limited data for HFC use during cardiac surgery in pediatric patients, we selected NS as the comparator in this study. Based on our results, a study randomizing patients to HFC or cryoprecipitate, instead of NS, would also be valuable. It should also be noted that the results of this study may have been influenced by the preoperative treatment with FFP in patients with an antithrombin III deficiency, or by the routine application of post-bypass platelets for all patients. Additionally, the range of surgical types included among the low number of patients in this study may have affected the results.

## Conclusion

HFC (70 mg/kg) administered after CPB termination was demonstrably efficacious and reduced the need for transfusion with cryoprecipitate without concomitant increases in AEs in this small sample size. In our experience, all infants have coagulopathies following CPB, which must be corrected. The ability to treat acquired hypofibrinogenemia in young infants with HFC instead of cryoprecipitate is advantageous due to the lower volumes required, and because it will theoretically reduce the incidence of blood-borne infections.

## Supplementary Information

Below is the link to the electronic supplementary material.

Supplementary file1 (DOCX 50 kb)

## Data Availability

Access to the full study protocol and study data can be requested from the corresponding author.
