# Supplementary material for: A Randomized Pilot Trial Assessing the Role of Human Fibrinogen Concentrate in Decreasing Cryoprecipitate Use and Blood Loss in Infants Undergoing Cardiopulmonary Bypass
Source: Pediatr Cardiol. 2022 Mar 19;43(7):1444–54. doi: 10.1007/s00246-022-02866-4 (PMC9489575; doi:10.1007/s00246-022-02866-4)
Supplement: Supplementary file 1 — Supplementary file1 (DOCX 50 kb) [file 246_2022_2866_MOESM1_ESM.docx]

# Supplementary Material

**Article title:** A randomized pilot trial assessing the role of human fibrinogen concentrate in decreasing cryoprecipitate use and blood loss in infants undergoing cardiopulmonary bypass

**Journal:** Pediatric Cardiology

**Authors:** Christopher F. Tirotta, Richard G. Lagueruela, Apeksha Gupta, Daria Salyakina, David Aguero, Jorge Ojito, Kathleen Kubes, Robert Hannan, Redmond P. Burke

**Corresponding author:** Christopher F. Tirotta, Dept. Anesthesiology, The Heart Program, Nicklaus Children’s Hospital; Email: christirotta@att.net

Supplementary Table 1. Pre-Defined Formulae for Dosing Amounts

| **Product** | **Dose** |
| --- | --- |
| PRBC | Volume required = blood volume (V) x desired hematocrit increase/hematocrit of PRBC (70%) |
| FFP | 20 cc/kg |
| Plateletpheresis | 20 cc/kg |
| Cryoprecipitate | 10 cc/kg |

FFP, fresh frozen plasma; PRBC, packed red blood cells.

Supplementary Table 2. ROTEM Analysis Algorithm

| **Four main scenarios based on clinical observation and ROTEM results were possible:** | |
| --- | --- |
| Insignificant bleeding—normal ROTEM | No transfusions |
| Insignificant bleeding—abnormal ROTEM | No transfusions |
| Significant bleeding—normal ROTEM | Surgical re-evaluation |
| Significant bleeding—abnormal ROTEM | Transfusion of blood products as indicated by:  a. FIBTEM MCF <7 mm 🡪 cryoprecipitate  b. HEPTEM MCF <50 mm 🡪 platelets  c. HEPTEM CT >240 seconds 🡪 FFP  d. HEPTEM CFT  >110 seconds 🡪 cryoprecipitate and/or platelets depending on MCF |

CFT, clot formation time; CT, clotting time; FFP, fresh frozen plasma; MCF, maximum clot firmness; ROTEM, rotational thromboelastometry.

## **Supplementary Table 3**. Baseline ROTEM Values

|  | **Overall** | | **Treatment** | | **Placebo** | |
| --- | --- | --- | --- | --- | --- | --- |
|  | **N** | **Median (IQR)** | **N** | **Median (IQR)** | **N** | **Median (IQR)** |
| **INTEM** | | | | | | |
| Baseline preop CT (sec) | 30 | 189.5 (168.0-212.0) | 15 | 182.0 (158.0-212.0) | 15 | 194.0 (178.0-218.0) |
| Baseline preop CFT (sec) | 30 | 67.5 (58.0-79.0) | 15 | 64.0 (56.0-77.0) | 15 | 73.0 (58.0-90.0) |
| Baseline preop α (°) | 30 | 77.0 (74.0-78.0) | 15 | 77.0 (75.0-79.0) | 15 | 75.0 (73.0-78.0) |
| Baseline preop A10 (mm) | 30 | 55.5 (51.0-58.0) | 15 | 57.0 (52.0-59.0) | 15 | 52.0 (51.0-58.0) |
| Baseline preop A20 (mm) | 30 | 60.5 (56.0-63.0) | 15 | 61.0 (58.0-65.0) | 15 | 58.0 (55.0-62.0) |
| Baseline preop MCF (mm) | 30 | 60.5 (57.0-63.0) | 15 | 61.0 (59.0-66.0) | 15 | 58.0 (56.0-62.0) |
| Baseline preop ML (%) | 30 | 5.5 (4.0-10.0) | 15 | 6.0 (4.0-10.0) | 15 | 5.0 (5.0-10.0) |
| **HEPTEM** | | | | | | |
| Baseline preop CT (sec) | 30 | 192.0 (170-214.0) | 15 | 190.0 (165.0-209.0) | 15 | 199.0 (173.0-234.0) |
| Baseline preop CFT (sec) | 30 | 67.5 (59.0-80.0) | 15 | 67.0 (57.0-71.0) | 15 | 73.0 (59.0-92.0) |
| Baseline preop α (°) | 30 | 77.0 (75.0-78.0) | 15 | 77.0 (76.0-78.0) | 15 | 75.0 (71.0-78.0) |
| Baseline preop A10 (mm) | 30 | 55.0 (50.0-58.0) | 15 | 56.0 (53.0-58.0) | 15 | 51.0 (49.0-58.0) |
| Baseline preop A20 (mm) | 30 | 59.0 (55.0-62.0) | 15 | 61.0 (57.0-64.0) | 15 | 56.0 (53.0-61.0) |
| Baseline preop MCF (mm) | 30 | 59.0 (55.0-62.0) | 15 | 61.0 (58.0-65.0) | 15 | 56.0 (53.0-61.0) |
| Baseline preop ML (%) | 30 | 6.0 (4.0-10.0) | 15 | 7.0 (4.0-10.0) | 15 | 6.0 (5.0-12.0) |
| **FIBTEM** | | | | | | |
| Baseline preop CT (sec) | 30 | 55.0 (52.0-62.0) | 15 | 55.0 (52.0-65.0) | 15 | 55.0 (48.0-62.0) |
| Baseline preop CFT (sec) | 2 | 153.0 (85.0-221.0) | 2 | 153.0 (85.0-221.0) | 0 | - |
| Baseline preop α (°) | 22 | 72.0 (66.0-76.0) | 10 | 74.0 (70.0-78.0) | 12 | 67.5 (62.5-73.0) |
| Baseline preop A10 (mm) | 30 | 12.0 (10.0-15.0) | 15 | 12.0 (10.0-16.0) | 15 | 12.0 (9.0-14.0) |
| Baseline preop A20 (mm) | 30 | 12.5 (10.0-16.0) | 15 | 12.0 (10.0-17.0) | 15 | 13.0 (9.0-14.0) |
| Baseline preop MCF (mm) | 30 | 12.5 (10.0-16.0) | 15 | 12.0 (10.0-17.0) | 15 | 13.0 (9.0-14.0) |
| Baseline preop ML (%) | 30 | 0.0 (0.0-0.0) | 15 | 0.0 (0.0-0.0) | 15 | 2.0 (0.0-5.0) |
| **EXTEM** | | | | | | |
| Baseline preop CT (sec) | 30 | 58.5 (54.0-66.0) | 15 | 59.0 (53.0-66.0) | 15 | 58.0 (54.0-67.0) |
| Baseline preop CFT (sec) | 30 | 82.5 (70.0-95.0) | 15 | 77.0 (70.0-87.0) | 15 | 87.0 (67.0-103.0) |
| Baseline preop α (°) | 30 | 73.0 (71.0-76.0) | 15 | 74.0 (72.0-76.0) | 15 | 72.0 (69.0-76.0) |
| Baseline preop A10 (mm) | 30 | 55.0 (51.0-59.0) | 15 | 57.0 (53.0-60.0) | 15 | 53.0 (50.0-58.0) |
| Baseline preop A20 (mm) | 30 | 60.5 (57.0-65.0) | 15 | 62.0 (60.0-65.0) | 15 | 58.0 (56.0-63.0) |
| Baseline preop MCF (mm) | 30 | 61.5 (58.0-65.0) | 15 | 62.0 (58.0-66.0) | 15 | 59.0 (57.0-65.0) |
| Baseline preop ML (%) | 30 | 7.0 (5.0-12.0) | 15 | 7.0 (6.0-12.0) | 15 | 7.0 (5.0-14.0) |

A10, amplitude at 10 minutes; A20, amplitude at 20 minutes; CFT, clot formation time; CT, clotting time; IQR, interquartile range; MCF, maximum clot firmness; ML, maximum lysis; op, operative.

Supplementary Table 4. Additional Post-Drug Clinical Characteristics Among Treatment and Placebo Groups

|  | **Overall** | | | **Treatment** | | **Placebo** | | |
| --- | --- | --- | --- | --- | --- | --- | --- | --- |
|  | **N** | **Median (IQR)** | **N** | | **Median (IQR)** | | **N** | **Median (IQR)** |
| 2 hours postop Hg (gm/dL) | 30 | 12.8 (11.8-13.9) | 15 | | 13.6 (11.0-15.2) | | 15 | 12.5 (11.7-13.3) |
| 2 hours postop Hct | 30 | 37.6 (32.7-41.0) | 15 | | 40.9 (32.7-43.5) | | 15 | 35.9 (35.4-38.8) |
| 2 hours postop PLT (10k/uL) | 30 | 272.0 (234.0-309.0) | 15 | | 282.0 (234.0-309.0) | | 15 | 224.0 (192.0-311.0) |
| 2 hours postop PT (sec) | 30 | 16.8 (16.7-17.2) | 15 | | 17.0 (16.7-18.5) | | 15 | 16.5 (15.8-17.2) |
| 2 hours postop INR | 30 | 1.4 (1.33-1.44) | 15 | | 1.4 (1.3-1.5) | | 15 | 1.4 (1.2-1.4) |
| 2 hours postop PTT (sec) | 30 | 35.8 (33.7-38.1) | 15 | | 36.7 (33.7-41.1) | | 15 | 35.2 (32.1-36.6) |
| 2 hours postop Fib (mg/dL) | 29 | 281.0 (238.0-353.0) | 15 | | 258.0 (238.0-338.0) | | 14 | 330.0 (281.0-385.0) |
| 24 hours postop Hg (gm/dL) | 30 | 12.9 (11.7-14.8) | 15 | | 13.8 (11.7-15.8) | | 15 | 12.4 (11.0-14.7) |
| 24 hours postop Hct | 30 | 38.3 (34.1-43.4) | 15 | | 38.9 (34.1-45.3) | | 15 | 35.4 (32.9-42.8) |
| 24 hours postop PLT (10k/uL) | 30 | 248.0 (197.0-297.0) | 15 | | 249.0 (197.0-297.0) | | 15 | 247.0 (189.0-300.0) |
| 24 hours postop PT (sec) | 28 | 16.1 (15.4-17.6) | 15 | | 16.0 (15.4-18.2) | | 13 | 16.1 (14.9-17.1) |
| 24 hours postop INR | 28 | 1.3 (1.2-1.4) | 15 | | 1.3 (1.2-1.5) | | 13 | 1.3 (1.2-1.4) |
| 24 hours postop PTT (sec) | 28 | 35.1 (34.1-37.4) | 15 | | 35.5 (34.1-38.6) | | 13 | 31.5 (30.3-35.5) |
| 24 hours postop Fib (mg/dL) | 28 | 416.5 (318.5-512.0) | 15 | | 427.0 (318.0-556.0) | | 13 | 403.0 (324.0-465.0) |
| Prime CPB PRBC (cc/kg) | 30 | 50.6 (44.7-61.3) | 15 | | 50.1 (44.7-62.0) | | 15 | 51.1 (39.0-61.3) |
| Prime CPB PLT (cc/kg) | 30 | 0.0 (0.0-0.0) | 15 | | 0.0 (0.0-0.0) | | 15 | 0.0 (0.0-0.0) |
| Prime CPB FFP (cc/kg) | 30 | 0.0 (0.0-0.0) | 15 | | 0.0 (0.0-0.0) | | 15 | 0.0 (0.0-0.0) |
| Run CPB PRBC (cc/kg) | 30 | 0.0 (0.0-0.0) | 15 | | 0.0 (0.0-0.0) | | 15 | 0.0 (0.0-0.0) |
| Run CPB PLT (cc/kg) | 30 | 46.0 (34.6-53.0) | 15 | | 42.4 (34.6-49.9) | | 15 | 50.2 (38.5-53.2) |
| Run CPB FFP (cc/kg) | 30 | 0.0 (0.0-0.0) | 15 | | 0.0 (0.0-0.0) | | 15 | 0.0 (0.0-0.0) |
| Total CPB (cc/kg) | 30 | 93.2 (80.2-113.1) | 15 | | 98.3 (80.2-113.1) | | 15 | 92.1 (82.9-113.8) |
| Anesthesia CS (cc/kg) | 30 | 21.6 (19.9-31.0) | 15 | | 21.5 (19.9-27.3) | | 15 | 23.2 (13.4-31.8) |
| Anesthesia FFP (cc/kg) | 30 | 0.0 (0.0-19.1) | 15 | | 0.0 (0.0-19.1) | | 15 | 0.0 (0.0-0.0) |
| Anesthesia PRBC (cc/kg) | 30 | 0.0 (0.0-0.0) | 15 | | 0.0 (0.0-0.0) | | 15 | 0.0 (0.0-0.0) |
| Anesthesia PLT (cc/kg) | 30 | 10.4 (0.4-14.5) | 15 | | 10.2 (0.0-12.8) | | 15 | 10.6 (0.0-21.5) |
| Anesthesia cryo (cc/kg)* | 30 | 2.1 (0.1-11.6) | 15 | | 0.0 (0.0-0.0) | | 15 | 11.0 (4.5-14.3) |
| Total Anesthesia blood (cc/kg) | 30 | 38.0 (21.9-68.3) | 15 | | 36.2 (21.9-55.1) | | 15 | 38.9 (31.0-70.2) |
| Anesthesia FVII (mcg/kg) | 30 | 0.0 (0.0-0.0) | 15 | | 0.0 (0.0-0.0) | | 15 | 0.0 (0.0-0.0) |
| ICU CS (cc/kg) | 30 | 0.0 (0.0-7.2) | 15 | | 0.0 (0.0-11.6) | | 15 | 0.0 (0.0-0.0) |
| ICU FFP (cc/kg) | 30 | 0.0 (0.0-0.9) | 15 | | 0.0 (0.0-0.9) | | 15 | 0.0 (0.0-0.0) |
| ICU PRBC (cc/kg) | 30 | 0.0 (0.0-0.0) | 15 | | 0.0 (0.0-0.0) | | 15 | 0.0 (0.0-0.0) |
| ICU PLT (cc/kg) | 30 | 0.0 (0.0-14.3) | 15 | | 0.0 (0.0-18.2) | | 15 | 0.0 (0.0-0.0) |
| ICU cryo (cc/kg) | 30 | 0.0 (0.0-0.0) | 15 | | 0.0 (0.0-0.0) | | 15 | 0.0 (0.0-0.0) |
| Total ICU blood (cc/kg) | 30 | 8.6 (0.6-30.9) | 15 | | 7.2 (0.0-39.7) | | 15 | 8.9 (0.0-28.5) |
| ICU FVII (mcg/kg) | 30 | 0.0 (0.0-0.0) | 15 | | 0.0 (0.0-0.0) | | 15 | 0.0 (0.0-0.0) |
| ICU EBL (cc/kg) | 30 | 34.8 (20.6-54.3) | 15 | | 35.2 (20.6-61.1) | | 15 | 34.4 (27.8-54.3) |
| Total FVII (mcg/kg) | 29 | 0.0 (0.0-0.0) | 14 | | 0.0 (0.0-0.0) | | 15 | 0.0 (0.0-0.0) |
| Total PRBC (cc/kg) | 30 | 56.6 (44.7-89.1) | 15 | | 55.3 (44.7-77.2) | | 15 | 57.9 (44.8-89.2) |
| Total FFP (cc/kg) | 30 | 0.0 (0.0-30.4) | 15 | | 0.0 (0.0-23.2) | | 15 | 13.6 (0.0-45.1) |
| Total PLT (cc/kg) | 30 | 59.9 (50.1-69.3) | 15 | | 61.3 (50.1-69.3) | | 15 | 59.9 (50.9-73.0) |
| Total cryo (cc/kg)* | 30 | 7.5 (0.5-12.3) | 15 | | 0.0 (0.0-0.0) | | 15 | 12.0 (8.2-14.3) |
| Total CS (cc/kg) | 30 | 25.2 (20.3-34.8) | 15 | | 27.3 (20.3-34.8) | | 15 | 23.7 (13.5-47.5) |
| Total blood (cc/kg) | 30 | 141.8 (137.6-208.3) | 15 | | 142.1 (137.6-174.8) | | 15 | 140.9 (136.2-219.3) |
| Int hrs | 30 | 11.5 (0.5-69.0) | 15 | | 0.0 (0.0-28.5) | | 15 | 24.0 (0.0-7.0) |
| LOS ICU (days)** | 29 | 8.0 (4.0-12.0) | 14 | | 8.0 (4.0-23.0) | | 15 | 8.0 (5.0-12.0) |
| LOS hospital (days)** | 29 | 9.0 (6.0-12.0) | 14 | | 9.0 (6.0-23.0) | | 15 | 8.0 (6.0-12.0) |

*significant at p<0.05; **excluded a patient with LOS 99 days

CPB, cardiopulmonary bypass; cryo, cryoprecipitate; CS, cell saver; EBL, estimated blood loss; FFP, fresh frozen plasma; Fib, fibrinogen; FVII, factor VII; Hct, hematocrit; Hg, hemoglobin; ICU, intensive care unit; INR, international normalized ratio; Int, intubation; IQR, interquartile range; LOS, length of stay; PLT, platelet; PRBC, packed red blood cells; PT, prothrombin time; PTT, partial thromboplastin time.

## **Supplementary Table 5**. Post-Drug Other ROTEM Results Among Treatment and Placebo Groups

|  | **Overall** | | **Treatment** | | **Placebo** | |
| --- | --- | --- | --- | --- | --- | --- |
|  | **N** | **Median (IQR)** | **N** | **Median (IQR)** | **N** | **Median (IQR)** |
| **HEPTEM** | | | | | | |
| Post-drug off bypass OR CT (sec) | 30 | 305.0 (255.0-421.0) | 15 | 328.0 (274.0-395.0) | 15 | 292.0 (221.0-540.0) |
| Post-drug off bypass OR CFT (sec) | 30 | 112.5 (93.0-150.0) | 15 | 114.0 (94.0-140.0) | 15 | 103.0 (85.0-298.0) |
| Post-drug off bypass OR α (°) | 30 | 68.5 (61.0-72.0) | 15 | 68.0 (65.0-71.0) | 15 | 70.0 (43.0-73.0) |
| Post-drug off bypass OR A10 (mm) | 29 | 45.0 (42.0-50.0) | 15 | 45.0 (43.0-50.0) | 14 | 46.5 (31.0-51.0) |
| Post-drug off bypass OR A20 (mm) | 28 | 54.0 (48.0-57.0) | 13 | 55.0 (52.0-57.0) | 15 | 53.0 (38.0-57.0) |
| Post-drug off bypass OR MCF (mm) | 30 | 55.5 (48.0-60.0) | 15 | 55.0 (53.0-60.0) | 15 | 56.0 (44.0-59.0) |
| 24 h post-op CT (sec) | 29 | 187.0 (173.0-205.0) | 15 | 191.0 (178.0-209.0) | 14 | 178.0 (161.0-192.0) |
| 24 h post-op CFT (sec) | 29 | 65.0 (57.0-82.0) | 15 | 69.0 (56.0-82.0) | 14 | 62.0 (57.0-89.0) |
| 24 h post-op α (°) | 29 | 77.0 (73.0-79.0) | 15 | 76.0 (73.0-79.0) | 14 | 77.5 (73.0-79.0) |
| 24 h post-op A10 (mm) | 29 | 60.0 (56.0-63.0) | 15 | 58.0 (55.0-63.0) | 14 | 62.0 (57.0-63.0) |
| 24 h post-op A20 (mm) | 29 | 64.0 (62.0-67.0) | 15 | 63.0 (60.0-66.0) | 14 | 65.0 (62.0-67.0) |
| 24 h post-op MCF (mm) | 29 | 64.0 (61.0-67.0) | 15 | 63.0 (60.0-67.0) | 14 | 65.0 (62.0-67.0) |
| 24 h post-op ML (%) | 29 | 6.0 (4.0-8.0) | 15 | 6.0 (4.0-10.0) | 14 | 6.5 (4.0-7.0) |
| **INTEM** | | | | | | |
| Post-drug off bypass OR CT (sec) | 30 | 303.5 (250.0-443.0) | 15 | 310.0 (266.0-403.0) | 15 | 272.0 (212.0-801.0) |
| Post-drug off bypass OR CFT (sec) | 30 | 109.0 (92.0-141.0) | 15 | 107.0 (90.0-130.0) | 15 | 139.0 (93.0-408.0) |
| Post-drug off bypass OR α (°) | 28 | 69.5 (63.5-72.0) | 15 | 69.0 (66.0-72.0) | 13 | 70.0 (62.0-72.0) |
| Post-drug off bypass OR A10 (mm) | 30 | 47.0 (43.0-51.0) | 15 | 47.0 (44.0-52.0) | 15 | 47.0 (27.0-51.0) |
| Post-drug off bypass OR A20 (mm) | 28 | 55.5 (49.0-58.5) | 13 | 57.0 (53.0-59.0) | 15 | 53.0 (39.0-58.0) |
| Post-drug off bypass OR MCF (mm) | 30 | 57.0 (49.0-61.0) | 15 | 58.0 (54.0-62.0) | 15 | 55.0 (45.0-60.0) |
| Post-drug off bypass OR ML (%) | 30 | 0.0 (0.0-1.0) | 15 | 0.0 (0.0-2.0) | 15 | 0.0 (0.0-1.0) |
| 24 h post-op CT (sec) | 29 | 199.0 (187.0-217.0) | 15 | 212.0 (190.0-221.0) | 14 | 189.5 (177.0-205.0) |
| 24 h post-op CFT (sec) | 29 | 67.0 (59.0-82.0) | 15 | 71.0 (58.0-82.0) | 14 | 63.5 (59.0-72.0) |
| 24 h post-op α (°) | 29 | 77.0 (74.0-78.0) | 15 | 75.0 (73.0-78.0) | 14 | 78.0 (75.0-78.0) |
| 24 h post-op A10 (mm) | 29 | 59.0 (56.0-63.0) | 15 | 58.0 (55.0-62.0) | 14 | 61.5 (58.0-63.0) |
| 24 h post-op A20 (mm) | 29 | 65.0 (62.0-67.0) | 15 | 63.0 (60.0-68.0) | 14 | 65.5 (63.0-67.0) |
| 24 h post-op MCF (mm) | 29 | 65.0 (63.0-67.0) | 15 | 64.0 (60.0-68.0) | 14 | 65.5 (63.0-67.0) |
| 24 h post-op ML (%) | 29 | 6.0 (4.0-8.0) | 15 | 5.0 (3.0-9.0) | 14 | 6.0 (4.0-7.0) |
| **EXTEM** | | | | | | |
| Post-drug off bypass OR CT (sec) | 30 | 84.0 (76.0-90.0) | 15 | 83.0 (76.0-88.0) | 15 | 87.0 (75.0-98.0) |
| Post-drug off bypass OR CFT (sec) | 30 | 117.5 (94.0-129.0) | 15 | 105.0 (93.0-122.0) | 15 | 126.0 (75.0-183.0) |
| Post-drug off bypass OR α (°) | 30 | 67.0 (65.0-71.0) | 15 | 69.0 (66.0-71.0) | 15 | 65.0 (58.0-69.0) |
| Post-drug off bypass OR A10 (mm) | 30 | 49.5 (43.0-53.0) | 15 | 51.0 (46.0-53.0) | 15 | 47.0 (40.0-50.0) |
| Post-drug off bypass OR A20 (mm) | 29 | 57.0 (51.0-60.0) | 14 | 58.0 (54.0-60.0) | 15 | 55.0 (48.0-58.0) |
| Post-drug off bypass OR MCF (mm) | 30 | 59.0 (54.0-62.0) | 15 | 61.0 (56.0-63.0) | 15 | 58.0 (51.0-62.0) |
| Post-drug off bypass OR ML (%) | 30 | 0.0 (0.0-1.0) | 15 | 0.0 (0,0-1.0) | 15 | 0.0 (0.0-1.0) |
| 24 h post-op CT (sec) | 29 | 66.0 (60.0-70.0) | 15 | 68.0 (61.0-72.0) | 14 | 65.0 (56.0-68.0) |
| 24 h post-op CFT (sec) | 29 | 77.0 (65.0-94.0) | 15 | 80.0 (65.0-94.0) | 14 | 74.0 (61.0-103.0) |
| 24 h post-op α (°) | 29 | 76.0 (71.0-77.0) | 15 | 75.0 (71.0-77.0) | 14 | 76.0 (69.0-78.0) |
| 24 h post-op A10 (mm) | 29 | 60.0 (57.0-65.0) | 15 | 57.0 (57.0-65.0) | 14 | 63.0 (55.0-66.0) |
| 24 h post-op A20 (mm) | 29 | 66.0 (63.0-69.0) | 15 | 64.0 (63.0-69.0) | 14 | 68.0 (60.0-70.0) |
| 24 h post-op MCF (mm) | 29 | 67.0 (63.0-69.0) | 15 | 65.0 (63.0-69.0) | 14 | 69.0 (60.0-70.0) |
| 24 h post-op ML (%) | 29 | 5.0 (4.0-9.0) | 15 | 5.0 (4.0-9.0) | 14 | 5.5 (4.0-7.0) |

A10, amplitude at 10 minutes; A20, amplitude at 20 minutes; CFT, clot formation time; CT, clotting time; IQR, interquartile range; MCF, maximum clot firmness; ML, maximum lysis; op, operative; OR, operating room.
